# Public health round-up

**DOI:** 10.2471/BLT.23.011123

**Published:** 2023-11-01

**Afghanistan earthquake**

Men searching for victims trapped in debris of a collapsed building in Naieb Rafi village, Zindajan district, Herat Province, Afghanistan, which was hit by earthquakes on 7, 11 and 15 October 2023. As of 11 October, the World Health Organization (WHO) and partners had reached communities in the worst-affected districts, providing assistance that included trauma and rehabilitation services and mental health and psychosocial support.

**Figure:**
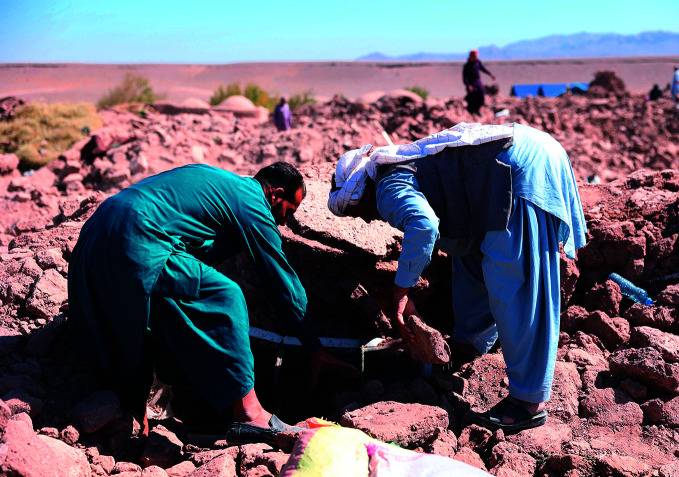
WHO/Zakarya Safari

## Conflict in Israel and the occupied Palestinian territory

An unprecedented attack on the civilian population of Israel by the terrorist organization Hamas which began on 7 October, sparked a massive retaliation on the Gaza Strip in the occupied Palestinian territory (oPt).

As of October 12, over 1200 Israeli and foreign nationals, the majority of whom civilians, were reported to have been killed and more than 3000 wounded. As of the same date, 1100 Palestinians were reported to have been killed, and more than 5000 injured. Those numbers were expected to rise.

As a result of a blockade imposed on Gaza by Israel, which included shutting off electricity and water, as of October 12, hospitals in the Gaza Strip were relying on back-up generators, with fuel set to run out in the following days. Supplies pre-positioned by WHO before the Hamas attack had been exhausted.

Access for emergency medical teams in the field was being severely hampered by infrastructure damage. The World Health Organization (WHO) documented 34 attacks on health care in Gaza between 7 and 12 October that resulted in the deaths of 11 health workers, and damage to 19 health facilities and 20 ambulances.

WHO called for an end to the hostilities and for participants in the conflict to allow health and humanitarian assistance to get through. WHO also expressed grave concern about the health and well-being of hostages, including elderly civilians, seized from Israel by Hamas in the 7 October attacks, calling for their health and medical needs to be addressed immediately, and for their safe release.

As of 12 October, WHO stood ready to dispatch trauma and essential health supplies from its logistics hub in Dubai in the United Arab Emirates.

https://bit.ly/3tqwRW9

https://bit.ly/3ZQ8hKw

https://bit.ly/3ZWhYa4

## Afghanistan earthquake

A 6.3 magnitude earthquake hit the western province of Herat, Afghanistan on 7 October. Another earthquake of the same magnitude struck Herat on 11 October, followed by at least three aftershocks.

As of 10 October, the United Nations Office for the Coordination of Humanitarian Affairs (OCHA) Afghanistan had reported 1294 deaths and 1688 injuries. Those numbers were expected to increase as rescue operations progressed and aftershocks continued.

As of 11 October, WHO had reported damage to a total of 21 health facilities across 10 affected districts.

WHO and 15 Health Cluster partners had reached 5625 individuals across three highly affected districts (Zindajan, Injil and Herat) in Herat Province, providing assistance that included trauma and rehabilitation services and mental health and psychosocial support.

https://bit.ly/48SuTxS

## A second malaria vaccine

WHO recommended a new vaccine for the prevention of malaria in children. The R21/Matrix-M vaccine is the second malaria vaccine recommended by WHO, following the RTS,S/AS01 vaccine, which received a WHO recommendation in 2021. Both vaccines have been shown to be safe and effective in preventing malaria in children, and are expected to have a significant public health impact.

The R21 vaccine will now complete the ongoing WHO prequalification process which, if successful, will enable international procurement of the vaccine for broad roll-out.

According to a 2 October media release, at least 28 countries in Africa plan to introduce a WHO-recommended malaria vaccine as part of their national immunization programmes. The RTS,S vaccine will be rolled out in some African countries in early 2024, and the R21 malaria vaccine is expected to become available to countries mid-2024.

WHO also issued recommendations on the advice of the Strategic Advisory Group of Experts (SAGE) for new vaccines for dengue and meningitis, along with immunization schedules and product recommendations for COVID-19. WHO also issued key immunization programmatic recommendations on polio.

https://bit.ly/46M6uZ3

## Funding boost for polio eradication

The European Commission, the European Investment Bank and the Bill & Melinda Gates Foundation announced a new financing partnership, committing 1.1 billion euros (€) (1.2 billion United States dollars) to achieving polio eradication and other critical global health goals.

Announced on 11 October, the expected financing package will comprise €500 million in new funding for the Global Polio Eradication Initiative; €500 million in investments and grants to boost access to health innovations, strengthen health systems and prepare for future pandemics; and €80 million in grants to provide technical assistance and ensure that global health programmes achieve their full potential.

https://bit.ly/3rLcjqZ

## Commitment to pandemic preparedness

The first heads of state summit on pandemic prevention, preparedness and response ended on 20 September at the United Nations General Assembly with a political declaration calling for international cooperation, coordination, governance and investment needed to better prepare the world for future pandemics.

Included in the declaration was an acknowledgment of the need for Member States to conclude negotiations on a WHO convention, agreement or other international instrument on pandemic prevention, preparedness and response, otherwise known as the Pandemic Accord, and continue their work to make targeted amendments to the International Health Regulations by May 2024.

https://bit.ly/48KW0Lv

## Surging cholera

Strong evidence of a significant surge in the 7^th^ global cholera pandemic was confirmed. According to WHO’s Weekly epidemiological record published 27 September, the global number of cholera cases reported to WHO doubled between 2021 and 2022 from 223 370 to 472 697, while the number of countries reporting cases rose from 35 to 44.

Current data for 2023 suggest that this global upsurge is continuing. Twenty-four countries are currently reporting active outbreaks, with some countries in the midst of acute crises.

https://bit.ly/3tuxkXm

## Mental health of refugees and migrants

WHO published a new report on the mental health of refugees and migrants. Released on 10 October, *Mental health of refugees and migrants: risk and protective factors and access to care* presents evidence regarding patterns of risk and protective factors, and of facilitators and barriers to care.

In related news, WHO and the Office of the High Commissioner on Human Rights jointly launched new guidance on mental health, human rights and legislation, to support countries to reform legislation in order to end human rights abuses and increase access to quality mental health care.

https://bit.ly/46D01Qe

https://bit.ly/3LZ0qV0

## Tackling postpartum haemorrhage

WHO released its first global roadmap to tackle postpartum haemorrhage (PPH) –excessive bleeding after childbirth – which affects millions of women annually, and is the world’s leading cause of maternal deaths, resulting in around 70 000 deaths per year.

Released on 11 October, the roadmap outlines goals and activities for research, normative work, implementation and advocacy, and aims to help countries address stark differences in survival outcomes from PPH, which reflect major inequities in access to essential health services. Over 85% of deaths from PPH occur in the African and South-East Asia Regions.

https://bit.ly/3rMXior

**Cover photo**

A malaria supervisor collects water from a fish hatchery in Lohandiguda in Bastar district, Chhattisgarh, India.

**Figure:**
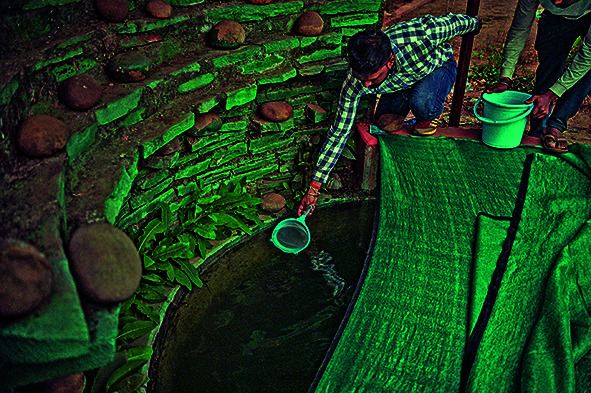
WHO/Atul Loke/Panos Pictures

## Premature births

An estimated 13.4 million babies were born early (before 37 full weeks of pregnancy) in 2020 – around 1 in 10 of all live births – according to a study by WHO, the United Nations Children’s Fund (UNICEF) and the London School of Hygiene and Tropical Medicine which was published in the Lancet on 5 October.

Prematurity is a leading cause of death in children, so there is an urgent need to strengthen both care for preterm babies as well as prevention efforts – particularly maternal health and nutrition – to improve childhood survival.

https://bit.ly/3LXbgv1

## Electronic prequalification

A platform for the digital processing of prequalification (PQ) information will be ready for launch in January 2024. According to a 27 September announcement, the electronic PQ System (ePQS) will allow manufacturers, national regulatory agencies and other stakeholders to log into a web portal, lodge applications and receive notification of pending actions.

In related news, the report of an independent assessment of WHO’s Department of regulation and prequalification (RPQ) was published on 28 September. Noting the significant impact RPQ has had in terms of enabling access to critical health products for the global population among other key findings, the report also sets out opportunities for future work.

https://bit.ly/3tyELwU

https://bit.ly/3ZUayUV

**Looking ahead**

6–8 November 2023. World Local Production Forum: The Hague, Kingdom of the Netherlands. https://bit.ly/3Fe0aOe

14–16 November 2023. World Health Innovation Forum: Visakhapatnam, India. https://bit.ly/46ERMUl

30 November–12 December 2023. COP28 Health Pavilion: Dubai, United Arab Emirates. https://bit.ly/3PP1k7L

